# Impact of Parental Cancer on Children: Differences by Child’s Age and Parent’s Disease Stage

**DOI:** 10.3390/children11060687

**Published:** 2024-06-04

**Authors:** Gessica Migliaccio, Michela Petrillo, Valentina Abate, Ornella Mazzoni, Daria Di Bonito, Daniela Barberio

**Affiliations:** Oncologic Psychology, Istituto Nazionale Tumori—IRCCS-Fondazione G. Pascale, 80131 Naples, Italy; gessica.migliaccio@istitutotumori.na.it (G.M.); michela.petrillo@istitutotumori.na.it (M.P.); v.abate@istitutotumori.na.it (V.A.); o.mazzoni@istitutotumori.na.it (O.M.); d.dibonito@istitutotumori.na.it (D.D.B.)

**Keywords:** parental cancer, communication, children, risk factors, psycho-oncology

## Abstract

(1) Receiving an oncological diagnosis involves countless physical and mental challenges for those who become ill. In addition to this, developmental activities are put on hold when a parent becomes ill, significantly impairing children’s normal growth and development. The purpose of this review is to highlight the psychological impact of cancer on children, with particular attention to differences based on the age of the child and the stage of the parent’s disease. (2) Articles published on PubMed up until October 2023 were searched. Qualitative and quantitative studies were included in this review after an evaluation of the full text. The study selection process was undertaken by two researchers, and articles for which there was unanimous agreement between researchers were included in the review. (3) Children’s psycho-emotional responses differ based on their age and the disease stage. In general, good communication and a supportive family environment that understands everyone’s needs seem to constitute important protective factors that favor the adaptation of the entire family to the disease. (4) The family, as an evolutionary system, finds itself facing phases typical of development. Knowing the variables that intervene in the process of adaptation to the disease will allow us to design specific and differentiated clinical interventions based on the needs of not only the patient but also the entire family.

## 1. Introduction

Receiving a cancer diagnosis is a traumatic event, and the patient may experience high levels of distress, anxiety, and depression. When a parent with dependent children is diagnosed with cancer, the diagnosis leads to changes throughout the family system [1,2,3,4]. The physical, role, daily routine organization, and economic changes related to the disease, among others, involve all family members as they are interconnected, directly and indirectly [5,6]. Sudden disruptions to daily life, financial stress, worsening physical conditions, and decreased emotional availability contribute to stress and uncertainty for parents and their families [1,7,8].

To evaluate whether parenting conditions influence parental distress levels, a study compared cancer patients with and without children. From the analysis of the results, it emerged that the group of patients with children (age < 18 years) recorded significantly higher levels of anxiety and depression [9]. As confirmed by other research, it would seem that these levels of distress are related to parental concerns, both practical and emotional [10]. The parent with cancer must learn to negotiate between being a patient and being a parent: two roles that can conflict; concern about one’s role as a parent seems to increase as the disease progresses [11]. One of the main concerns of parents is the perception of their parental efficiency, understood as physical and emotional availability, which can be affected by frequent and repeated medical examinations and intense treatments that patients must constantly undergo [12,13,14]. 

Such medical tests and treatments can make patients less physically and emotionally available to their families. A study conducted on cancer survivors with minor and young adult children highlights how a reduced availability towards one’s family can decrease levels of trust in one’s parenting skills, becoming a major concern [10]. 

Living with cancer poses unique challenges for parents and their families. On the one hand, the parent worries and tries to protect their children from the consequences of the illness on their emotional development [15], but on the other hand, fatigue, pain, and loss of mobility can make them less available towards them, with a consequent reduction in the attention that was previously reserved for them [16,17]. Even if the healthy parent must redefine their role, they very often assume more responsibilities than before [15].

Parental diagnoses, the threat of death, and changes in daily routine therefore represent stress factors for children. This emotional experience may be a trigger of PTSD symptoms [18] and long-term psychosocial outcomes for children [19,20]. Parents’ anxious attitudes are likely to negatively influence parenting (e.g., overprotective behavior), which, in turn, may cause more anxiety and worry in children [4].

For adolescents, parental cancer disrupts typical developmental tasks, such as independence from parents, increased attention to body image, and peer group membership [5]. Although parental cancer affects children differently based on age, they are all known to be predisposed to countless developmental problems.

Therefore, this analysis of the existing literature, with particular attention to studies conducted on cancer patients with children aged between 0 and 22 years, aims to highlight any risk and protective factors at both the individual and family levels. We want to understand the impact of the disease on the emotional, social, and behavioral functioning of children. It is hypothesized that differences in adaptation to the disease will be attributable to the age of the children and the stage of the parent’s disease. The ultimate aim is to organize the work already present in the field in order to increase knowledge and identify and differentiate intervention strategies in clinical practice, based on the needs of the patient and their families.

## 2. Materials and Methods

**Figure 1:**
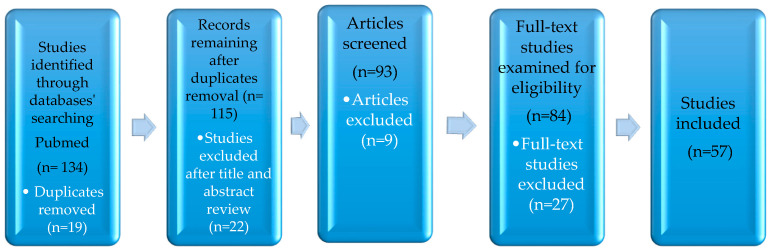
Flow diagram for the study selection process.

An extensive literature review was performed using the PubMed database, searching for articles published from inception to October 2023, using the words parental cancer; communication; children; risk factors; and psycho-oncology. A total of 134 documents were identified, and after removing duplicates, the remaining documents (*n* = 115) were screened for eligibility. Through a review of the title and abstract, 93 studies were retrieved and further examined. After an evaluation of the full-text articles, 57 studies were included in this review (see Figure 1). The study selection process was undertaken by two researchers, and articles for which there was unanimous agreement between researchers were included in the review.

## 3. Results

The ability to cope with stressful events is mediated by the emotional, cognitive, and environmental resources available. Illness represents a highly stressful event, and the ability to adapt may depend on both individual factors, which are different for each person, and factors linked to the illness or to the developmental stage of the child and family members. The results of studies in the field on cancer patients with children have been divided by the age of the child, the stage of the disease, risk and protection factors, and communication.

### 3.1. Differences Based on Children’s Age

When a parent is diagnosed with cancer, children may experience difficulties in coping, which are expressed in different symptoms based on age (Table 1).

Younger children, who do not yet possess full emotional awareness, may have difficulty accepting and managing negative emotions [18]. This difficulty can express itself with internalizing symptoms and post-traumatic stress disorder [3,22] or can increase the risk of depression. Some studies have shown that a worse prognosis and more intensive treatment are associated with more problems in children [24,25]. A parental cancer diagnosis can disrupt their daily routine due to medical examinations and hospitalization [12,17,26], and can affect children based on age. Preschoolers who rely on positive family functioning exhibit distress [2,21] and problems sleeping [7,21,23]. Meanwhile, school-age children express guilt because they feel responsible for the parent’s illness and are more moved by visible changes in the parent with cancer, such as hair loss and frequent vomiting [3,22,23].

Although children are considered more damageable and fragile, many studies have reported that adolescents are the most vulnerable to parent’s health problems [7,22]. It is understood that parental cancer produces greater distress in adolescents than in preschoolers [3,27]. Indeed, studies demonstrate that adolescents experience greater anxiety, depression, and distress than school-age children [19,22]. 

This difference can be explained by adolescents’ increased cognitive abilities, aptitude for abstract thought, greater awareness of their parent’s future physical conditions [28], and empathy, enabling them to be more conscious of losses and of the parent’s physical and emotional pain [24,29]. Indeed, in one study, adolescents reported feeling intense sadness, guilt, and concerns about impending parental death [23,29,30], showing empathy for a parent and awareness about the medical complications of cancer [11]. An association has been demonstrated between lower family cohesion and an increase in externalizing problems in adolescents [5].

A parent’s clinical condition can induce children, regardless of age, to accept more responsibilities within the family system and to detract from their involvement in extracurricular activities and relationships with peers [28,31,32,33]. Although parental cancer causes countless problems, the children of affected parents have reported that cancer implicates an increase in the time spent within the family and a closer proximity between family members. Therefore, cancer begins an opportunity to share and develop functional strategies to maintain relationships after the parent’s death [11].

### 3.2. Illness Stage 

The psychosocial impact of parental cancer on children may change based on the stage of the disease the parent is facing (Table 2). 

The results reported in the literature agree that the initial phase of the parent’s illness exposes children to a greater risk of developing internalizing disorders. However, no major psychosocial problems have been found. 

The terminal phase of the disease appears to be the period of greatest vulnerability for the patient and their family, sometimes even more than the period following death [35,37]. In fact, parents report social and behavioral problems in their children during this period [23,34]. However, a review published in 2007 reported no differences in the levels of distress experienced by children of terminally ill parents and a reference group. The variables consistently associated with the psychosocial development of children appear to be family communication, the parent’s adaptation to the illness, and their emotional experience [7].

Having advanced cancer leads to a greater awareness of one’s health and the limitations imposed by cancer [11]. On the one hand, this awareness exposes parents to a greater risk of psychological distress [37]; on the other hand, it motivates them to make a greater investment in parenting and in the relationship with their children [38]. One of the main concerns of parents with advanced cancer is the effects of their disease on their children and their own lives [32,39]. Studies comparing the children of healthy parents with those of parents suffering from advanced cancer highlight that it is the latter who experience the greatest discomfort. Some children and adolescents living with parents with advanced cancer show significantly higher levels of anxiety and depression and significantly lower self-esteem than their peers [34,35,36]. 

When cancer becomes chronic, the patient must face new challenges, such as living with the disease and accepting the constant presence of the disease and the uncertainty of the future. The ability to cope with information about the chronic nature of cancer depends on the amount of time available to patients and their families [15].

### 3.3. Risk and Protection Factors

Identifying risk and protection factors is advantageous for containing cancer’s impact on the physical and psychological wellness of patients and their families and for intervening in the most appropriate way. According to some studies, parental psychological problems are the most coherent predictors of a child’s psychological adaptation to parental illness [40]. In particular, a positive association was found between parental depression symptoms and children’s internalizing symptoms [41] and between PTSD symptoms in parents and children [4]. Parental depressive moods are associated with emotional and behavioral problems and inappropriate psychosocial functioning in children [40]. Many authors maintain that maternal depression and an inadequate and unclear definition of family roles increase the probability of internalizing problems, especially in daughters [5]. The studies also suggest that the possible factors that influence adolescents’ adaptation to parental illness are lacking parenthood, communication style, parental parenthood [23,36,38,42], and psychosocial functioning [4]. Meanwhile, it has been demonstrated that a good relationship between parents and children is a protective factor that can attenuate the effects of parental cancer diagnosis [43,44]. Furthermore, honest communication, cohesion, expressiveness, adaptability, reducing conflicts [45,46], and strategies to cope help to reduce stress and anguish in children and adolescents [12,47,48]. Considering the entire family system and its functioning, some studies have shown that it is possible to predict children’s symptoms that emerge at the family level, starting with an evaluation of the general functioning of the family [18]. In general, families with good communication report having closer relationships, which allows adolescents to better adjust to difficult life events [49,50]. Better family functioning determines better adaptation of the child [40]; therefore, it was interesting to identify the family characteristics that protect children from the emotional consequences of the parent’s cancer. The protective factors are a supportive family environment, mutual understanding between members, and low levels of conflict [18]. Another fundamental aspect concerns the benefit that adolescents derive from the support of peers experiencing the same situation [51].

#### Communication

The difficulties and concerns of parents regarding communication with their children concern both the identification of the best way to share emotionally charged information [52,53] and understanding how much, and if it is appropriate, to discuss abstract topics, such as death [37]. Research shows that the way parents communicate with their children may depend on the coping strategies they adopt [54]. Parents decide to communicate their diagnosis and prognosis to their children when they can understand and accept the possibility of their own death, when there is a worsening of the disease, a dramatic physical deterioration, and changes in treatments, and there are unexpected hospitalizations that impose changes and interruptions to the daily routine. Some parents, instead, reported that their children’s perceived emotional distress, together with a sense of violating parental protective instincts, made it difficult to engage in open communication with their children relative to diagnosis and prognosis [37]. The fear of not being able to answer the questions that the children might ask can lead to communication difficulties [15]. To protect their children, parents find it difficult to communicate with them and wish to avoid dealing, in particular, with the topic of death [55,56]. A study involving parents with advanced cancer shows a strong association between communication with their children, with respect to the disease, and high levels of anxiety. This correlation can be explained by the fact that those who had spoken openly with their children understood the possibility of death; this had not only prompted them to communicate with their children but had also contributed to the onset of a state of anxiety due to the understanding of their own prognosis [37]. It has been shown that poor communication between parents and children is associated with the onset of internalizing and externalizing problems [4]. Indeed, the fear that children feel regarding the recurrence of the disease or the parent’s death can manifest itself through somatic symptoms [5]; open family communication, however, is associated with lower levels of emotional and behavioral problems [45,57]. Communication has a central role in managing the effects of cancer on the entire family system; assessing its quality helps predict the child’s overall functioning in these cases [45,58]. Indeed, open communication between parents and children not only strengthens the relationship but also leads to the adoption of more effective coping strategies [23]. Although the difficulties experienced by patients who are both “sick” and “parents” are clear, doctors often prefer not to ask about them to avoid facing a painful topic or because they believe that parenting difficulties are too private. In this sense, individual and family psychological counseling could be a great resource for patients and their families, giving them a listening space to face and control their concerns [15] but also to help them identify a functional way to control the emotional and family situation.

## 4. Discussion

This work was born with the aim of reviewing current knowledge on the impact of a parent’s cancer on their children. The goal was to understand whether there were differences in children’s adaptation to their parent’s illness. The hypothesis that guided the interest in this work is that different levels of adaptation to parental illness depend both on the severity of the illness and on the emotional and cognitive developmental stages of the child. This interest arises from the observation, in clinical practice, of the different needs of patients, which often seem linked to these two factors. 

Some of the research analyzed supported this hypothesis by highlighting age-related differences in children’s psycho-emotional response: young children have more sleep problems [7,21,23], anguish [12,21], and sense of guilt [3,22,23]; adolescents suffer more from anxiety, depression, and anguish [19,22]. It may be that older adolescents, who are presumably more sensitive, are more aware of their parents’ feelings and distress than younger children. This increased burden appears to be expressed primarily in behavioral problems [4].

Regardless of age, however, there is a disinvestment in social relationships, preferring ever greater physical and emotional participation in family dynamics [28,31,32,33]. 

Other research, however, seems to highlight that the differences may be linked more to sex than to age, especially in the case of adolescents. It seems that daughters are more affected by their parent’s illness [4]. 

In this regard, we wonder whether these data can be traced back to a cultural stereotype that often sees females as more involved in tasks related to taking care of the home and their parents, with a consequent disinvestment from the typical developmental tasks of their age group.

With respect to the stage of the disease, some studies indicate that in the initial phase, children do not present greater psychosocial problems than the reference groups but do show an increased risk of developing them [7]. It can be assumed that in this phase of illness, the family’s attention is more focused on practical aspects, putting the emotional aspect on standby. When a parent is in an advanced stage of illness, greater levels of anxiety and depression are recorded [34,35,36]. These data can probably be traced back to the uncertainty of the future and the discomfort linked to the changes that the disease brings. The terminal phase seems to be the most complex [23,34], which is likely due to awareness of the end of life. This awareness can be experienced either with a prevalence of feelings of anguish over death or with a greater investment in relationships and improvement in the quality of the parent–child relationship.

However, the need for support seems to emerge to help parents communicate with their children in all phases of the disease, albeit in different ways [32]. Communication seems to be one of the main elements that can act as both a protective and risk factor in the processes of adaptation to the disease [4,46,57]. Overall, the most consistent predictor of a child’s psychological adjustment appears to be the mental health of the parents. Young children would be more affected by their parents’ emotional disorders, such as depressed moods [40]. Adolescents are more influenced by relational aspects, such as parenting style and communication. A favorable family environment, good cohesion, open and clear communication, and adaptive coping strategies are protective factors for both children and adolescents, and for the entire family in general [45,46]. Communication characterized by clarity, trust, and availability allows the creation of an authentic and reliable relationship that allows even negative emotions to be welcomed, giving them a different, more adaptive meaning.

## 5. Conclusions

Given the complexity of the topic and the lack of information and guidelines, we believe that future research is necessary for the definition of structured interventions to encourage the adaptation of patients and their families to the disease. Specific interventions would be useful for working preventively for the psycho-emotional health of cancer patients and their children.

## Figures and Tables

**Table 1:** Effects of a parent’s cancer diagnosis on their children based on age.

		Age	
Outcomes	0–6	7–12	12–18
	Anguish [12,21]	Sense of guilt [3,22,23]	Anxiety [19,22]
	Sleep problems [7,21,23]		Depression [19,22]
			Emotional distress [19,22]

**Table 2:** Relationship between children’s emotional response and disease stage.

Illness Stage	Results and Authors’ Conclusions
Early	Do not experience serious psychosocial difficulties Greater risk of problems [7]
Terminal	Behavioral and social problems as reported by parents [21,34]
Advanced cancer	Significantly high levels of anxiety and depression Significantly low levels of self-esteem [34,35,36]

## Data Availability

Not applicable.
